# Supplementary material for: Polarisome scaffolder Spa2-mediated macromolecular condensation of Aip5 for actin polymerization
Source: Nat Commun. 2019 Nov 7;10:5078. doi: 10.1038/s41467-019-13125-1 (PMC6838200; doi:10.1038/s41467-019-13125-1)
Supplement: Supplementary file 1 — Supplementary Information [file 41467_2019_13125_MOESM1_ESM.pdf]

Supplementary Information

Polarisome scaffolder Spa2-mediated macromolecular condensation of Aip5 for actin polymerization

(Ying et al.,)

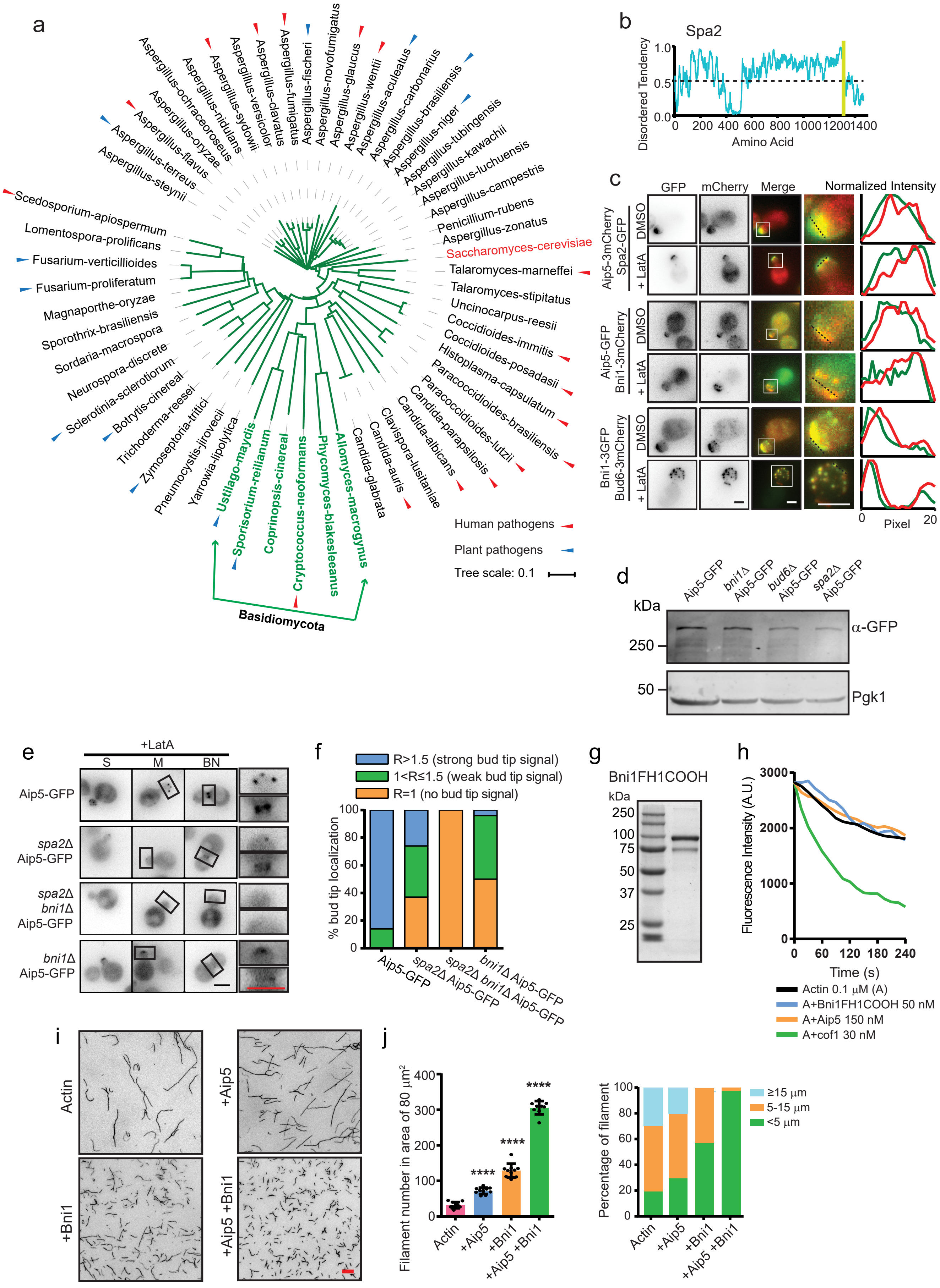

a
Saccharomyces-cerevisiae
Basidiomycota
Human pathogens
Plant pathogens
Tree scale: 0.1
b
Spa2
Disordered Tendency
Amino Acid
c
GFP
mCherry
Merge
Normalized Intensity
Aip5-3mCherry Spa2-GFP
Aip5-GFP Bni1-3mCherry
Bni1-3GFP Bud6-3mCherry
DMSO
+ LatA
Pixel
d
Aip5-GFP
bni1Δ Aip5-GFP
bud6Δ Aip5-GFP
spa2Δ Aip5-GFP
kDa
α-GFP
Pgk1
e
+LatA
S
M
BN
Aip5-GFP
spa2Δ Aip5-GFP
spa2Δ bni1Δ Aip5-GFP
bni1Δ Aip5-GFP
f
R>1.5 (strong bud tip signal)
1<R≤1.5 (weak bud tip signal)
R=1 (no bud tip signal)
% bud tip localization
g
Bni1FH1COOH
kDa
h
Fluorescence Intensity (A.U.)
Time (s)
Actin 0.1 μM (A)
A+Bni1FH1COOH 50 nM
A+Aip5 150 nM
A+cof1 30 nM
i
Actin
+Aip5
+Bni1
+Aip5 +Bni1
j
Filament number in area of 80 μm²
Percentage of filament
≥15 μm
5-15 μm
<5 μm

Supplementary figure 1. Aip5 protein analysis and *in vivo* localization.

(a) Aip5 protein sequence conservation analysis among species, the highlighted species in green color belongs to phylum Basidiomycota, the rest of the species belongs to phylum Ascomycota. **(b)** Predicted intrinsic disorder profile of Spa2 by ANCHOR. **(c)** Representative maximum Z-projection images of fluorescent fusions of polarisome proteins at the bud tip, in the absence or presence of 10 µM LatA for 30 min. Lines crossing 20 pixels were drawn at the bud tip in determining the colocalized fluorescence signals of GFP and mCherry. Scale bars represent 2 µm. (S, small-budded cell; M, medium size-budded cell; BN, bud neck.) **(d)** Western blot detection of endogenously expressed Aip5-GFP protein under a native promoter in WT and polarisome mutants. (Pgk1 was used as a loading control.) **(e)** Representative maximum Z-projection fluorescence images of Aip5-GFP at the but tip of WT and polarisome protein mutants, with a 10 µM LatA treatment for 30 min before imaging. Scale bars represent 3 µm. **(f)** Relative ratio (R) quantification of the signal intensity of Aip5-GFP at medium-sized bud tip in the conditions of (e). Aip5-GFP, n=49 cells; *spa2*Δ Aip5-GFP, n=35 cells; *spa2*Δ *bni1*Δ Aip5-GFP, n=19 cells; *bni1*Δ Aip5-GFP, n=26 cells. **(g)** SDS-PAGE of purified Bni1FH1COOH. **(h)** Time course of pyrene actin de-polymerization in the presence of full-length Aip5, Bni1FH1COOH, and Cof1, at the indicated protein concentration. **(i)** Representative fluorescence images of Acti-stain 488 phalloidin labeled-actin filaments that are polymerized from 2 µM actin, with the addition of 2 µM Aip5 for 30 min in the presence or absence of 0.5 µM Bni1FH1COOH. Scale bar represents 5 µm. **(j)** Quantification of the number and length of the actin filament in an area size of 80 μm^2. (control, 318 filaments from 10 fields; +Aip5, 389 filaments from 10 fields; +Bni1FH1COOH, 438 filaments from 10 fields; +Aip5+Bni1FH1COOH, 328 filaments from 9 fields) Source data are provided as a Source Data file.

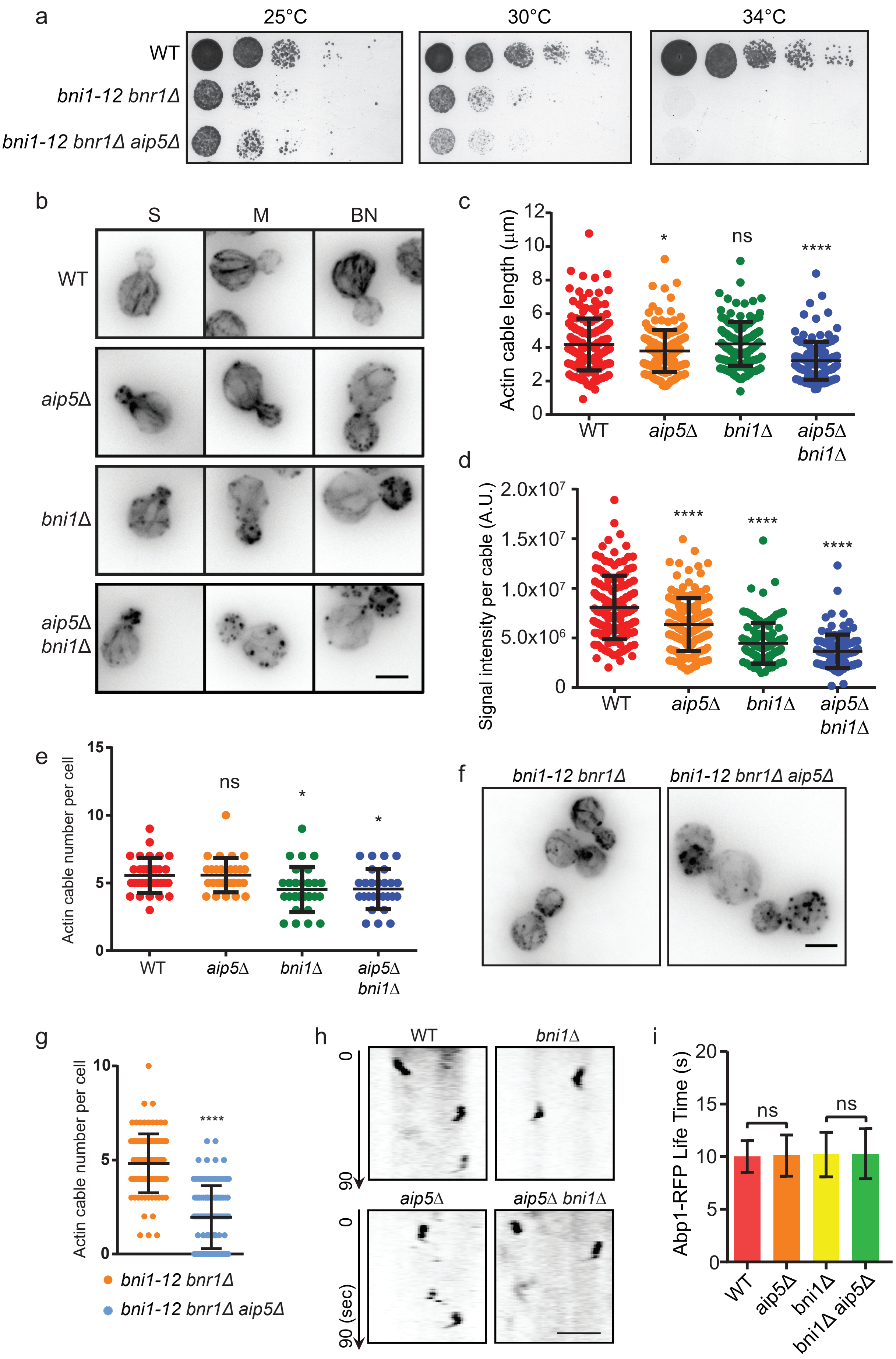

a
25°C
30°C
34°C
WT
bni1-12 bnr1Δ
bni1-12 bnr1Δ aip5Δ
b
S
M
BN
WT
aip5Δ
bni1Δ
aip5Δ bni1Δ
c
Actin cable length (μm)
12
10
8
6
4
2
0
*
ns
****
WT
aip5Δ
bni1Δ
aip5Δ bni1Δ
d
Signal intensity per cable (A.U.)
2.0x10^7
1.5x10^7
1.0x10^7
5.0x10^6
0
****
****
****
WT
aip5Δ
bni1Δ
aip5Δ bni1Δ
e
Actin cable number per cell
15
10
5
0
ns
*
*
WT
aip5Δ
bni1Δ
aip5Δ bni1Δ
f
bni1-12 bnr1Δ
bni1-12 bnr1Δ aip5Δ
g
Actin cable number per cell
10
5
0
****
bni1-12 bnr1Δ
bni1-12 bnr1Δ aip5Δ
h
WT
bni1Δ
aip5Δ
aip5Δ bni1Δ
0
90
0
90 (sec)
i
Abp1-RFP Life Time (s)
20
15
10
5
0
ns
ns
WT
aip5Δ
bni1Δ
bni1Δ aip5Δ

Supplementary figure 2. Aip5 and Bni1 regulate *in vivo* actin cable assembly.

(a) Genetic interaction of *AIP5* with *BNI1* was shown by spotting assay at the indicated temperatures after 36 hours growth. **(b)** Representative maximum Z-projection fluorescence images of Abp140-3GFP marked actin cables of the indicated strains. S, small-budded cell; M, medium size-budded cell; BN, bud neck. **(c, d)** Quantification of the length and total fluorescent intensity of Abp140-3GFP labelled actin cables (length: n=178 actin cable for wild type (WT); n=162 actin cable for *aip5*Δ; n=131 actin cable for *bni1*Δ; n=123 actin cable for *aip5*Δ *bni1*Δ. For actin cable intensity: n=149 actin cable for WT; n=153 actin cable for *aip5*Δ; n=106 actin cable for *bni1*Δ; n=112 actin cable for *aip5*Δ *bni1*Δ.) **(e)** Quantification of actin cable number labeled by Abp140-3GFP, as in (b). (WT, n=32 cells; *aip5*Δ, n=29 cells; *bni1*Δ, n=29 cells; *aip5*Δ *bni1*Δ, n=27 cells.) **(f)** Representative maximum Z-projection fluorescence images of Abp140-3GFP marked actin cables of the indicated strains at 25°C. **(g)** Quantification of detectable of actin cable, as in (f). (*bni1-12 bnr1*Δ, n=93 cells; *bni1-12 bnr1*Δ *aip5*Δ, n=102 cells) **(h)** Kymograph of Abp1-RFP lifetime from a movie of 90 seconds (1 frame/s), in the indicated strains. **(i)** Quantification of the Abp1-RFP lifetime, as in (h). (n=75 patches for each strain). All scale bars represent 3 µm. P-values in (c), (d), and (e) were determined by the one-way ANOVA, P-value in (g) was determined by the two-tailed Student's *t*-test assuming equal variances, ****p < 0.0001; *p < 0.05; ns = not significant. All the graphs represent mean ± S.D. Source data are provided as a Source Data file.

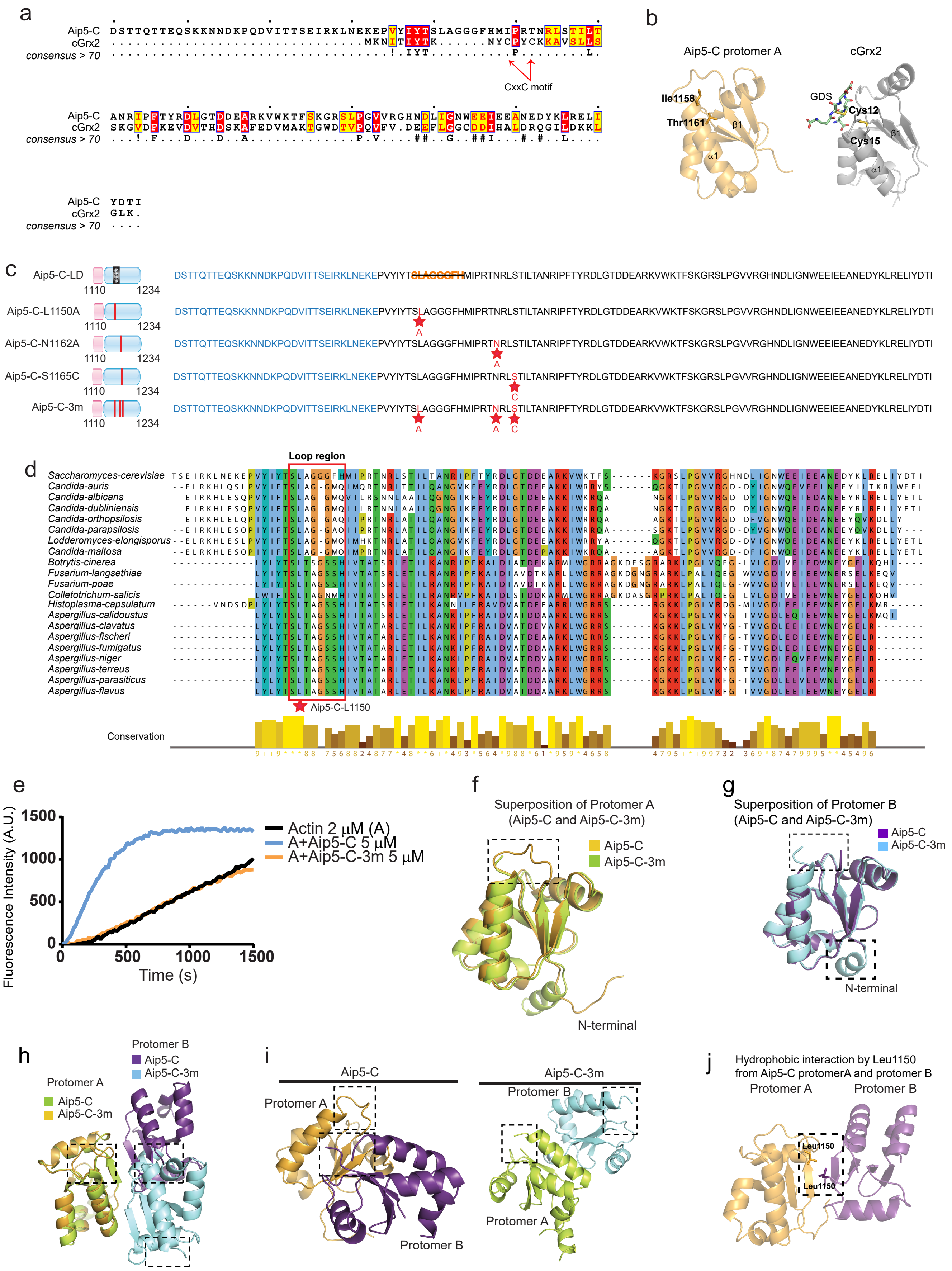

a
Aip5-C
cGrx2
consensus > 70
CxxC motif
b
Aip5-C protomer A
cGrx2
Ile1158
Thr1161
β1
α1
GDS
Cys12
Cys15
c
Aip5-C-LD
Aip5-C-L1150A
Aip5-C-N1162A
Aip5-C-S1165C
Aip5-C-3m
1110
1234
DSTTQTTEQSKKNNDKPQDVITTSEIRKLNEKEPVYIYTSLAGGGFHMIPRTNRLSTILTANRIPFTYRDLGTDDEARKVWKTFSKGRSLPGVVRGHNDLIGNWEEIEEANEDYKLRELIYDTI
d
Loop region
Saccharomyces-cerevisiae
Candida-auris
Candida-albicans
Candida-dubliniensis
Candida-orthopsilosis
Candida-parapsilosis
Lodderomyces-elongisporus
Candida-maltosa
Botrytis-cinerea
Fusarium-langsethiae
Fusarium-poae
Colletotrichum-salicis
Histoplasma-capsulatum
Aspergillus-calidoustus
Aspergillus-clavatus
Aspergillus-fischeri
Aspergillus-fumigatus
Aspergillus-niger
Aspergillus-terreus
Aspergillus-parasiticus
Aspergillus-flavus
Aip5-C-L1150
Conservation
e
Fluorescence Intensity (A.U.)
Time (s)
Actin 2 μM (A)
A+Aip5-C 5 μM
A+Aip5-C-3m 5 μM
f
Superposition of Protomer A
(Aip5-C and Aip5-C-3m)
Aip5-C
Aip5-C-3m
N-terminal
g
Superposition of Protomer B
(Aip5-C and Aip5-C-3m)
Aip5-C
Aip5-C-3m
N-terminal
h
Protomer A
Aip5-C
Aip5-C-3m
Protomer B
Aip5-C
Aip5-C-3m
i
Aip5-C
Protomer A
Protomer B
Aip5-C-3m
Protomer B
Protomer A
j
Hydrophobic interaction by Leu1150
from Aip5-C protomerA and protomer B
Protomer A
Protomer B
Leu1150
Leu1150

Supplementary figure 3. The homologous analysis of Aip5-C and Aip5-C mutant structure.

(a) Sequence alignment of Aip5-C with the structural homolog cGRx2, in which the CxxC motif for redox activity is absent in Aip5. **(b)** Structural comparison of Aip5-C with cGrx2 at the corresponding CxxC motif. **(c)** Domain schematics of Aip5-C mutation variants and the amino acid sequences. The deleted loop region and the sites of single mutations were highlighted. **(d)** Sequence alignment of Aip5-C among pathogenic filamentous fungus, illustrated by Jalview using the Clustal Omega. **(e)** Pyrene actin polymerization reaction with 5 µM Aip5-C or Aip5-C-3m. **(f)** Superposition of protomer A of Aip5-C and that of Aip5-C-3m. **(g)** Superposition of protomer B from Aip5-C and Aip5-C-3m. **(h)** Superposition of Aip5-C and Aip5-C-3m. A significant conformational change was observed upon mutations. Loop region that consists of L1150A mutation is highlighted in the black squared box. **(i)** The orientation of the loop region in Aip5-C and Aip5-C-3m. **(j)** Leu1150 in the loop connecting β1 and α1 in protomer A of Aip5-C forms hydrophobic interaction with Leu1150 from protomer B, stabilizing the dimer formation. Such establishment disappeared in Aip5-C-3m due to its conformational change upon mutations.

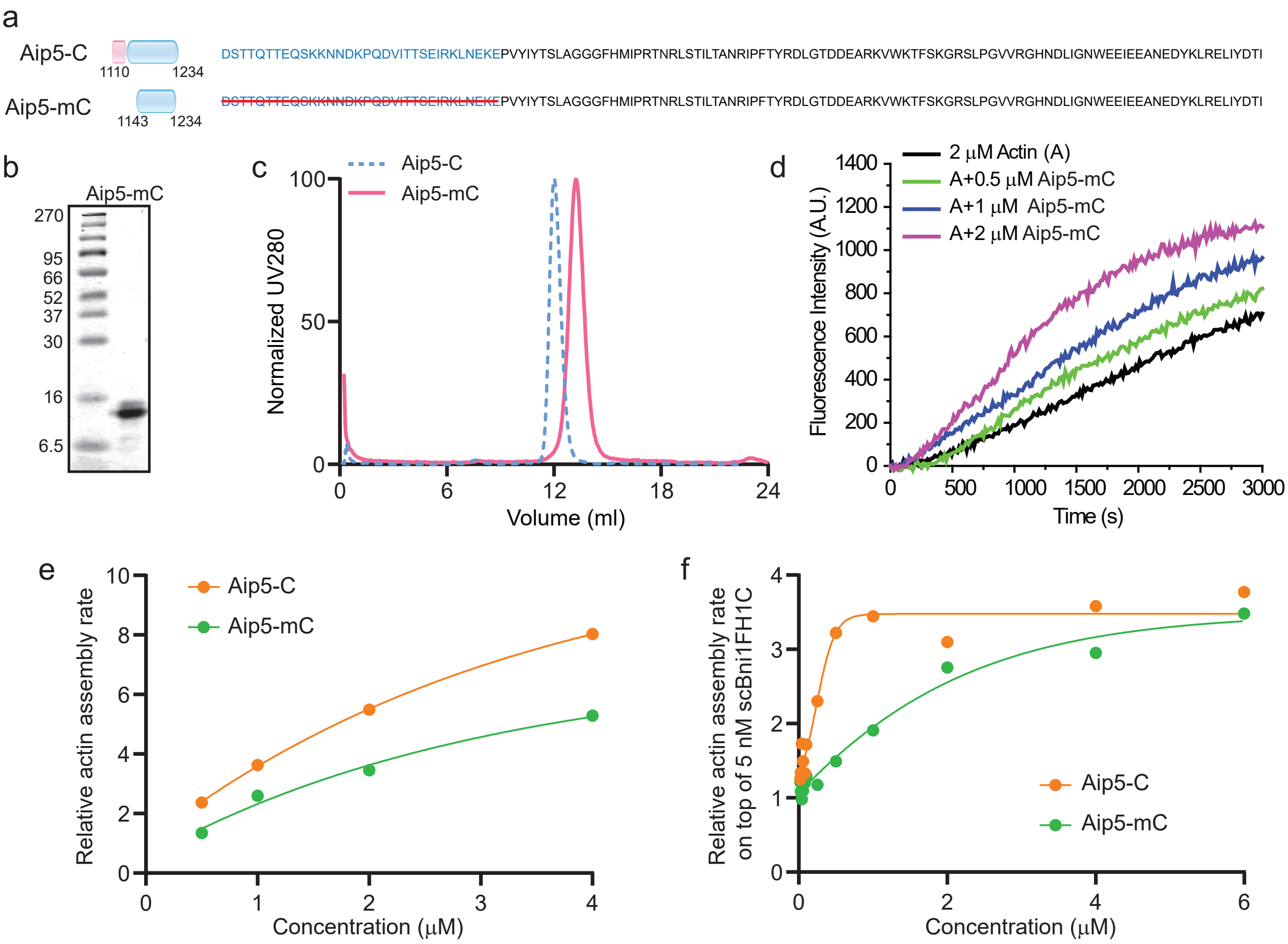

Supplementary figure 4. Monomeric Aip5-mC activities in actin polymerization.
(a) Domain schematics of dimer Aip5-C and monomeric version Aip5-mC in budding yeast, where the 33 residues at the N terminus were truncated and resulted in a monomer form of Aip5-C. **(b)** SDS-PAGE of purified monomeric Aip5-C protein in budding yeast (Aip5-mC). **(c)** The elution profile of Aip5-C and Aip5-mC proteins from calibration column Superdex 75 10/300 GL. **(d)** Pyrene assay with increasing concentrations of Aip5-mC. **(e)** Relative actin assembly rate of Aip5-C and Aip5-mC at the indicated concentrations by normalizing to the control of 2 μM actin. **(f)** Relative actin assembly rate of Aip5-C and Aip5-mC at the indicated concentrations by normalizing to 5 nM Bni1FH1COOH. The plots in (e) and (f), respectively, show the mean value from two biological replicates. Source data are provided as a Source Data file.

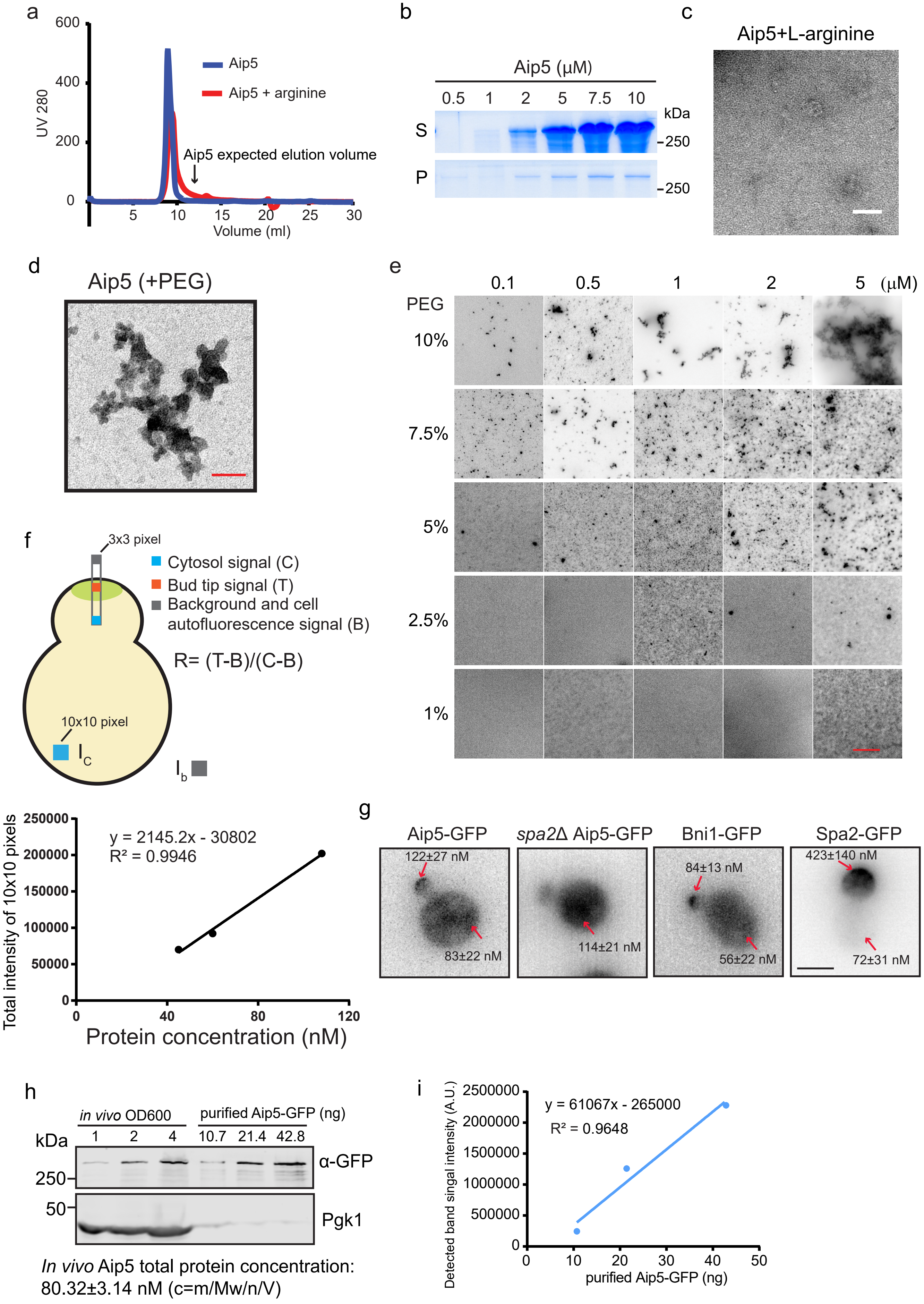

a
UV 280
Aip5
Aip5 + arginine
Aip5 expected elution volume
Volume (ml)
b
Aip5 (μM)
0.5 1 2 5 7.5 10
kDa
S
P
250
250
c
Aip5+L-arginine
d
Aip5 (+PEG)
e
0.1 0.5 1 2 5 (μM)
PEG
10%
7.5%
5%
2.5%
1%
f
3x3 pixel
Cytosol signal (C)
Bud tip signal (T)
Background and cell autofluorescence signal (B)
R= (T-B)/(C-B)
10x10 pixel
I_C
I_b
y = 2145.2x - 30802
R² = 0.9946
Total intensity of 10x10 pixels
Protein concentration (nM)
g
Aip5-GFP
spa2Δ Aip5-GFP
Bni1-GFP
Spa2-GFP
122±27 nM
83±22 nM
114±21 nM
84±13 nM
56±22 nM
423±140 nM
72±31 nM
h
in vivo OD600
1 2 4
purified Aip5-GFP (ng)
10.7 21.4 42.8
kDa
250
50
α-GFP
Pgk1
In vivo Aip5 total protein concentration:
80.32±3.14 nM (c=m/Mw/n/V)
i
y = 61067x - 265000
R² = 0.9648
Detected band singal intensity (A.U.)
purified Aip5-GFP (ng)

Supplementary figure 5. *In vitro* molecular assembly of Aip5 and *in vivo* Aip5 concentration.

(a) The elution profile of Aip5 from superdex 200 increase calibration column, with or without a pre-incubation with 500 nM of L-arginine for 15 min, where the Aip5 predicted dimer elution volume was indicated. **(b)** Soluble Aip5 protein, at a concentration in a range from 0.5 to 10 µM, from high-speed centrifugation for 30 min at 100,000g before subjecting to SDS-PAGE gel and staining. S: supernatant protein fraction; P: pellet protein fraction. **(c)** Representative TEM images of *in vitro* 1 µM full-length Aip5 incubating with 500 nM L-arginine on ice for 15 min before loading onto the grid. Scale bar represents 100 nm. **(d)** Representative TEM images of Aip5 assemblies *in vitro* at 1 µM formed at 5 min after incubating with 10% PEG3,350. The scale bar represents 100 nm. **(e)** Representative fluorescence images of Aip5 assemblies (10% Aip5-GFP) formed *in vitro* at the indicated concentrations, which were incubated with the indicated concentrations of PEG 3,350 for 5 min before imaging. The scale bar represents 5 µm. **(f)** *In vivo* concentration of polarisome proteins using signal intensity measurements, which were compared with reference proteins (see Methods). The signal intensities of the cytosol (I_c), background and auto-fluorescence intensity from cells (I_b), and bud tip (T) of medium size-budded cells were measured. The protein concentrations standard curve was generated. **(g)** The calculated polarisome proteins concentrations at bud tip and cytosol regions were indicated. The scale bar represents 3 µm. **(h)** Western blot detection of endogenously expressed Aip5-GFP protein under a native promoter in wild-type yeast and the purified recombinant protein Aip5-GFP. Pgk1 was used as a loading control. **(i)** The standard curve was generated by plotting Aip5-GFP protein mass against the protein signal intensity from western-blot, where the calculated total Aip5-GFP protein concentration *in vivo* was 80.32±3.14 nM. The value was mean and S.D calculated from three independent loading samples. Source data are provided as a Source Data file.

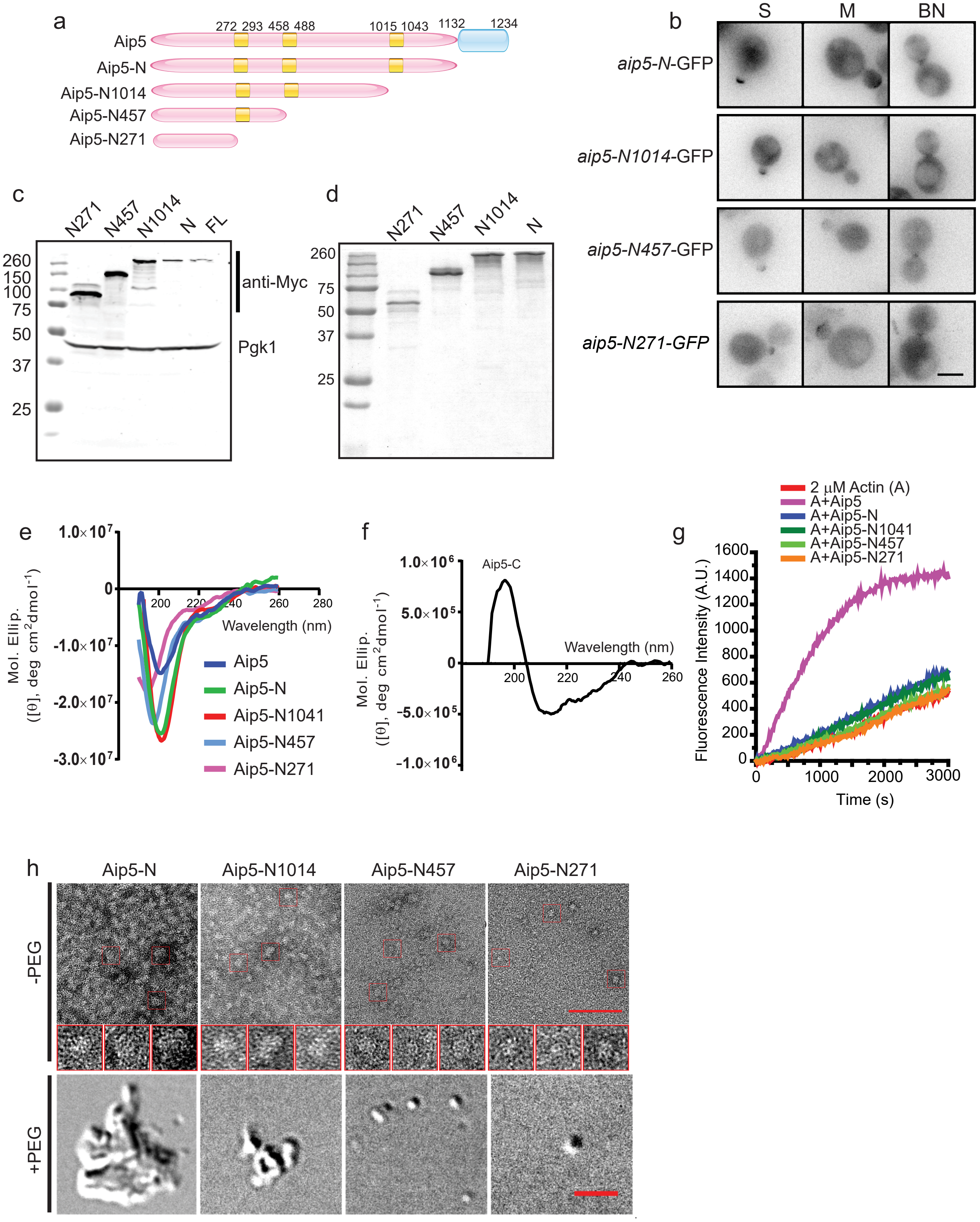
a
Aip5
Aip5-N
Aip5-N1014
Aip5-N457
Aip5-N271
272 293 458 488
1015 1043 1132 1234
b
S M BN
aip5-N-GFP
aip5-N1014-GFP
aip5-N457-GFP
aip5-N271-GFP
c
N271 N457 N1014 N FL
260 150 100 75 50 37 25
anti-Myc
Pgk1
d
N271 N457 N1014 N
260 75 50 37 25
e
Mol. Ellip. ([θ], deg cm2dmol-1)
Wavelength (nm)
Aip5
Aip5-N
Aip5-N1041
Aip5-N457
Aip5-N271
f
Aip5-C
Mol. Ellip. ([θ], deg cm2dmol-1)
Wavelength (nm)
g
2 μM Actin (A)
A+Aip5
A+Aip5-N
A+Aip5-N1041
A+Aip5-N457
A+Aip5-N271
Fluorescence Intensity (A.U.)
Time (s)
h
Aip5-N Aip5-N1014 Aip5-N457 Aip5-N271
-PEG
+PEG

Supplementary figure 6. N-terminal IDR determines the molecular condensation of Aip5.

(a) Domain schematics of Aip5. The Aip5 N terminal truncation proteins were designed based on the predicted coiled-coil domains using Pfam. **(b)** Representative maximum Z-projection images of endogenous GFP tagged Aip5 N terminal truncation proteins localization in yeast. S, small-budded cell; M, medium-size budded cell; BN, bud neck. Scale bar represents 3 µm. **(c)** Endogenously expressed Aip5 variants with the C-terminal Myc-tags that were detected by anti-Myc antibodies. (Pgk1 was used as the loading control.) **(d)** The SDS-PAGE gel of purified Aip5-N variants. **(e, f)** Circular dichroism spectra of Aip5 truncating variants at a concentration of 0.5 mg/ml. **(g)** Spontaneous actin polymerization by 2 µM Aip5 N-terminal truncations. **(h)** Representative TEM images of *in vitro*-formed condensates of Aip5 variants under non-PEG environments (upper panel). Insets showed an enlarged view of Aip5 oligomers. Differential interference contrast (DIC) images showed the protein condensates formed under 10% PEG 3,350 at 5 min (2 µM, lower panel). The scale bars represent 100 nm and 3 µm, respectively, for upper and lower panel.

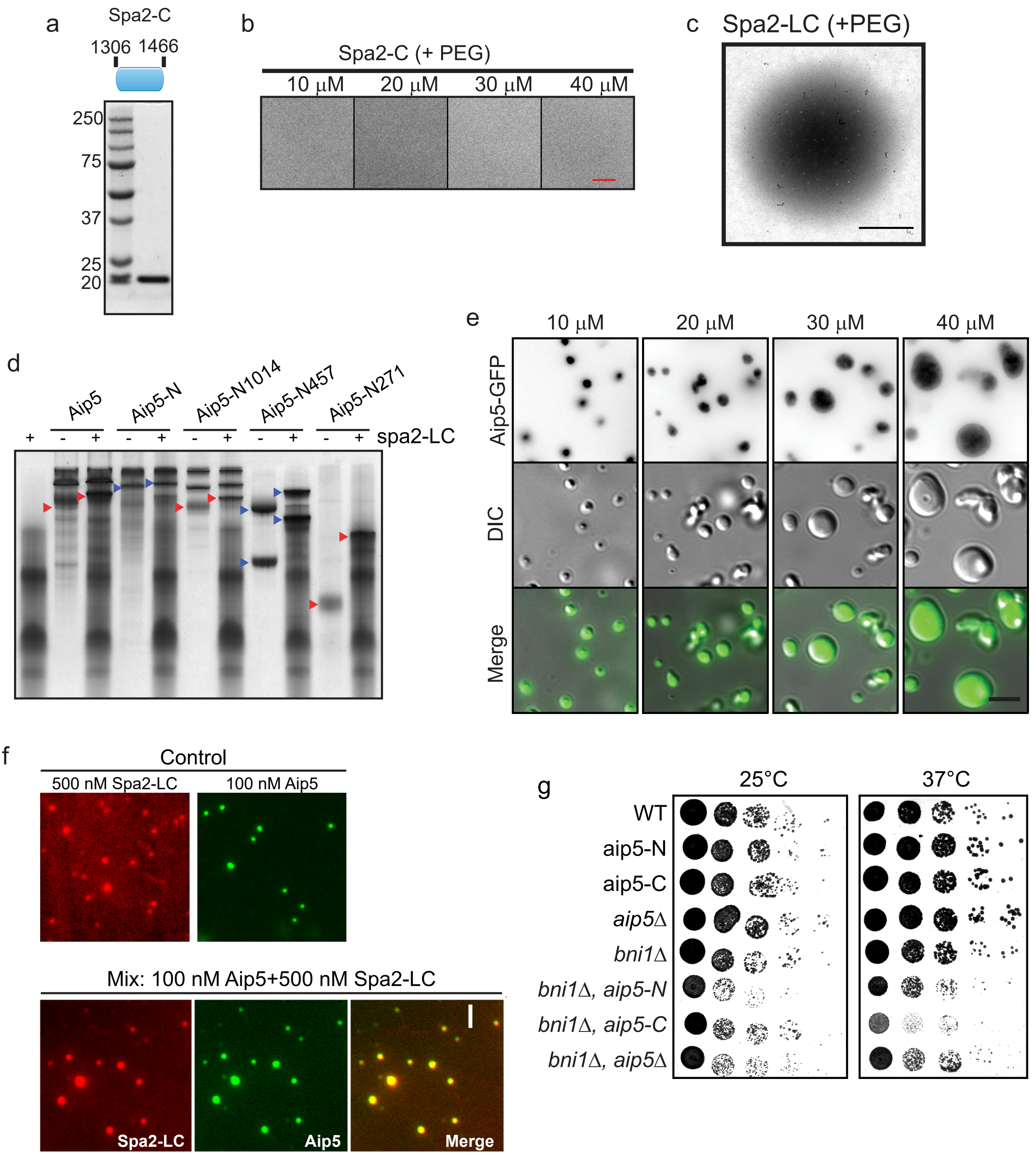

Supplementary figure 7. Liquid-liquid phase separation of Spa2 relies on its IDR region. **(a)** Domain schematic and SDS-PAGE gel of recombinant Spa2-C protein. **(b)** Representative DIC images of *in vitro* formation of Spa2-C assemblies in the presence of 10% PEG3,350 after 5 min of incubation at room temperature. Scale bar: 5 μm. **(c)** Representative TEM images of *in vitro*-formed Spa2-LC droplet (40 μM), which was induced by 10% PEG3,350 after 5 min of incubation at room temperature. Scale bar, 500 nm. **(d)** Native-PAGE gel of different protein combinations as indicated, where 4 μg of Spa2-LC was incubated with 2 μg of truncating Aip5 variants at room temperature for 30 min prior to electrophoresis. Arrows indicated the shifted bands of Aip5 variants in the presence of Spa2-LC. Native gel marker was not used. **(e)** Representative fluorescence images of *in vitro* formed droplets of Aip5 (500 nM, 10% Aip5-GFP) by mixing with the indicated concentrations of Spa2-LC for 5 min in 10% PEG3,350. The scale bar represents 5 μm. **(f)** Representative fluorescence images of 100 nM Aip5 (10% Aip5-GFP) and 500 nM Spa2-LC (10% Spa2-LC labelled by dye alexa568) formed protein assemblies under 20% peg10,000 at room temperature for 5 min. The scale bar represents 2 μm. **(g)** Yeast growth in spotting assay after 36 hours of incubation at 25°C and 37°C, respectively.

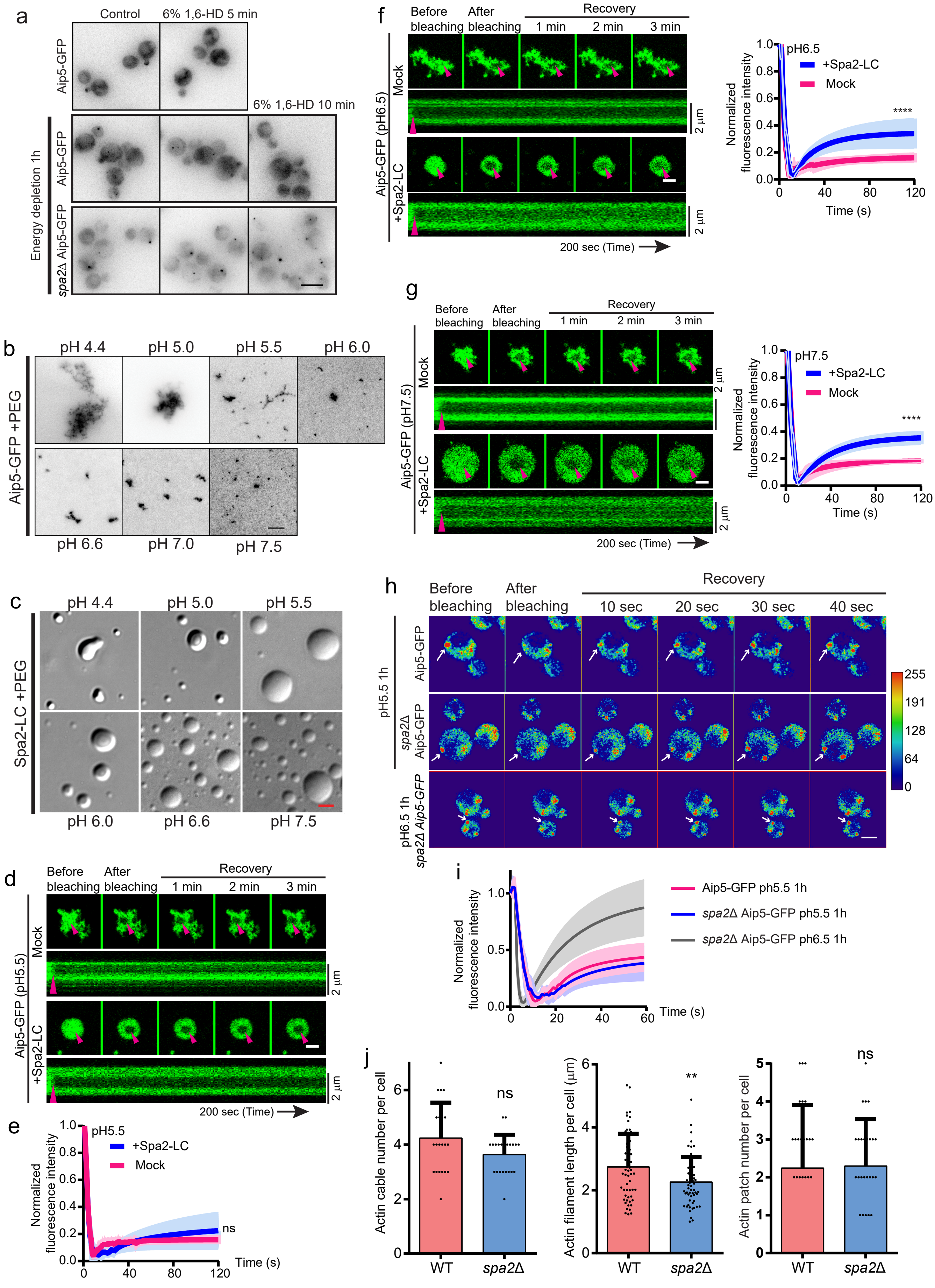
a
Control
6% 1,6-HD 5 min
6% 1,6-HD 10 min
Aip5-GFP
Energy depletion 1h
spa2Δ Aip5-GFP
b
Aip5-GFP +PEG
pH 4.4
pH 5.0
pH 5.5
pH 6.0
pH 6.6
pH 7.0
pH 7.5
c
Spa2-LC +PEG
d
Before bleaching
After bleaching
Recovery
1 min
2 min
3 min
Aip5-GFP (pH5.5)
Mock
+Spa2-LC
2 μm
200 sec (Time)
e
pH5.5
+Spa2-LC
Mock
ns
Normalized fluorescence intensity
Time (s)
f
Aip5-GFP (pH6.5)
pH6.5
****
g
Aip5-GFP (pH7.5)
pH7.5
h
10 sec
20 sec
30 sec
40 sec
pH5.5 1h
pH6.5 1h
spa2Δ Aip5-GFP
255
191
128
64
0
i
Aip5-GFP ph5.5 1h
spa2Δ Aip5-GFP ph5.5 1h
spa2Δ Aip5-GFP ph6.5 1h
j
Actin cable number per cell
Actin filament length per cell (μm)
Actin patch number per cell
WT
spa2Δ
**

Supplementary figure 8. Spa2-mediated Aip5 condensates *in vivo* under stressed conditions.

(a) Representative fluorescence images of Aip5-GFP signal in normal growth or 1-hour energy depletion condition treated by 6% 1, 6 hexanediol for 5 and 10 min. The scale bar represents 5 µm. **(b)** Representative fluorescence images of *in vitro* Aip5 (2 µM, 10% Aip5-GFP) assemblies that were formed in the indicated pH condition with 10% PEG3,350 for 5 min before imaging. The scale bar represents 5 µm. **(c)** Representative DIC images of *in vitro* Spa2-LC (30 µM) droplets formed in the indicated pH condition with 10% PEG3,350 for 5 min before imaging. The scale bar represents 2 µm. **(d-g)** Fluorescence recovery after photobleaching (FRAP) assay for *in vitro* Aip5 assemblies, in the absence or presence of Spa2-LC in three pH buffer (5.5, 6.5 and 7.5). ROIs used for generating kymographs were indicated by the arrowhead. 500 nM Aip5 (10% Aip5-GFP) was mixed with or without 30 µM Spa2-LC in the presence of 10% PEG 3,350. The scale bars represent 2 µm. Quantification of fluorescent signals of ROIs in FRAP experiments: n=7 for Aip5-GFP pH5.5; n=8 for Aip5-GFP+Spa2-LC pH5.5; n=8 for Aip5-GFP pH6.5; n=9 for Aip5-GFP+Spa2-LC pH6.5; n=8 for Aip5-GFP pH7.5; n=9 for Aip5-GFP+Spa2-LC pH7.5. **(h)** *In vivo* FRAP analysis of Aip5 condensates in wild-type and *spa2Δ* cells that were stressed by different pH buffer for 1-hour. Representative deconvoluted-images of Aip5 condensates, indicated by the white arrow, were shown. The colour key indicates signal intensity. The scale bar represents 5 µm. **(i)** Quantification of fluorescent signals of FRAP experiments, as in (h). n=12 ROIs for Aip5-GFP pH5.5 treatment; n=13 for *spa2*Δ Aip5-GFP pH5.5 treatment; n=15 for *spa2*Δ Aip5-GFP pH5.5 treatment. **(j)** Quantification of the Abp140-3GFP marked actin cables and actin patches under normal growth condition in Fig. 6b. Actin cable number: n=21 cells for WT; n=22 cells for *spa2Δ*; Actin filament length: n=57 cells for WT and *spa2Δ*; Actin patch number: n=29 cells for WT and n=27 cells for *spa2Δ*. P-values were determined by the two-tailed Student's *t*-test assuming equal variances, ****p < 0.0001, **p< 0.01. ns=not significant. All the bar graphs represent mean with an error bar, S.D. Source data are provided as a Source Data file.

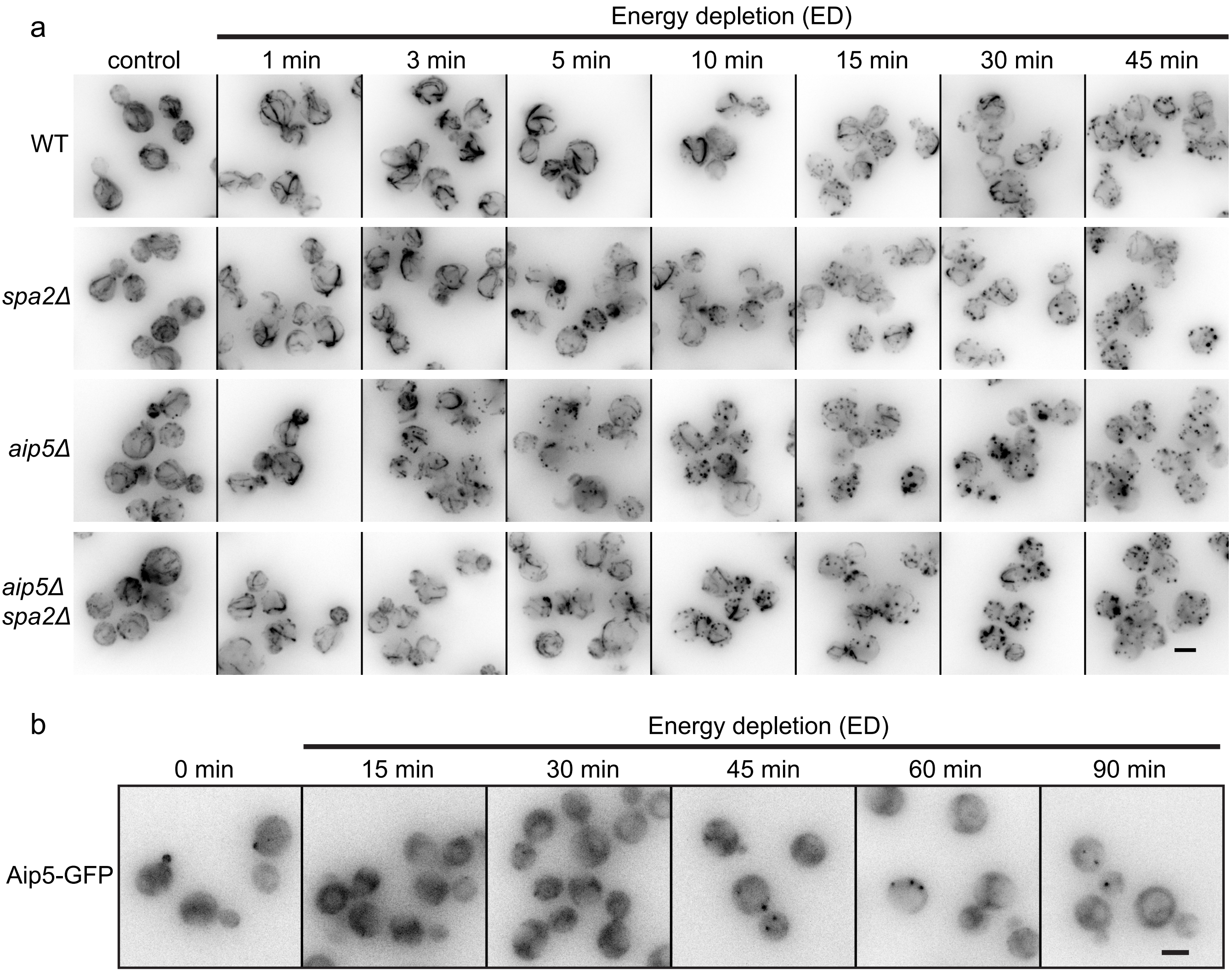

Supplementary figure 9. Time course phenotype of actin cable and Aip5 condensates under energy depletion condition.
(a) Representative fluorescence images of actin cable phenotypes revealed by Abp140-3GFP in the indicated yeast strains at different time points with energy depletion treatment (SD medium without glucose and supplement with 20 mM 2-Deoxy-D-glucose, 10 μM antimycin A). **(b)** Representative fluorescence images of Aip5-GFP aggregates formation in wild type at the indicated time points under energy depletion treatment. WT: wild type. Both scale bars represent 3 μm.

Supplementary figure 10.
Western blot and staining gel images displayed in the main and other supplementary figures. Red boxes indicate areas used for the figures.

Fig. 1c

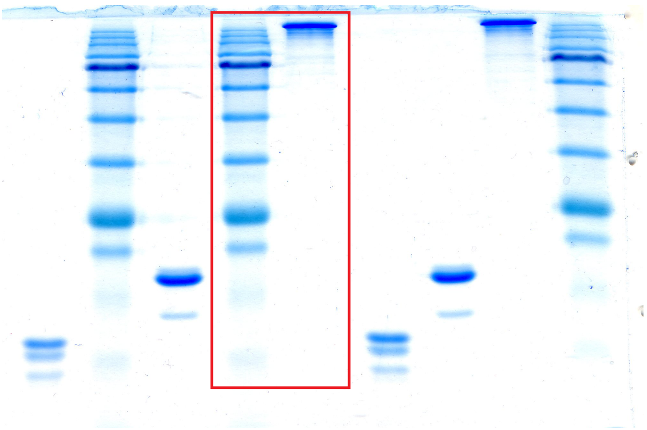

Fig. 2c

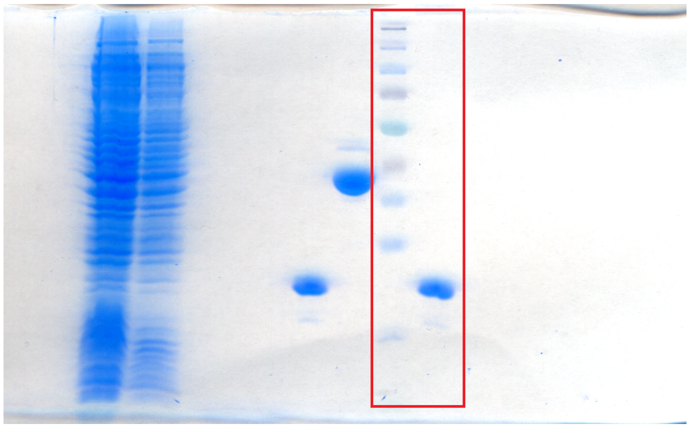

Fig. 2k

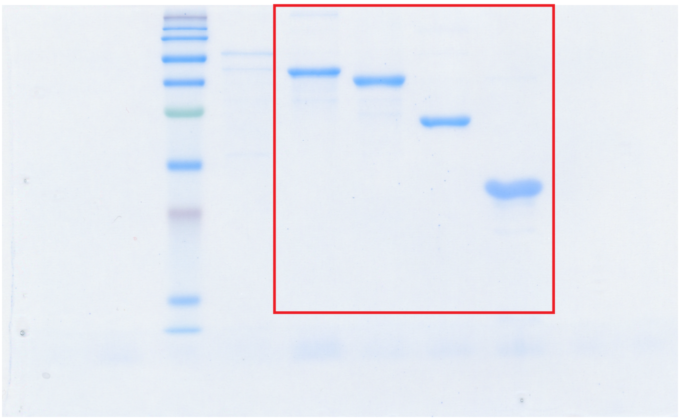

Fig. 4g

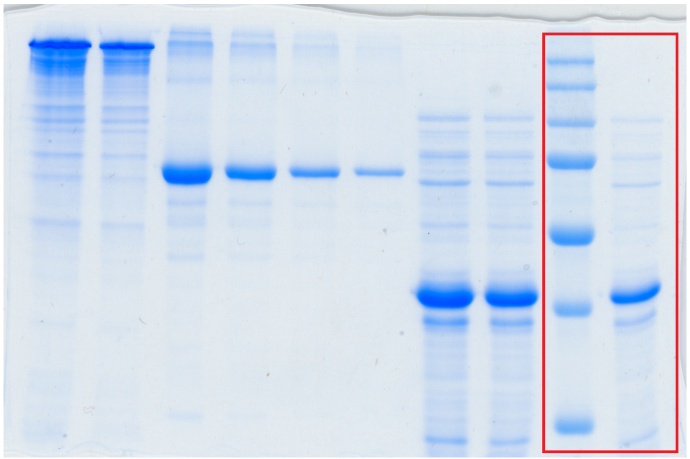

Supplementary Fig. 1d

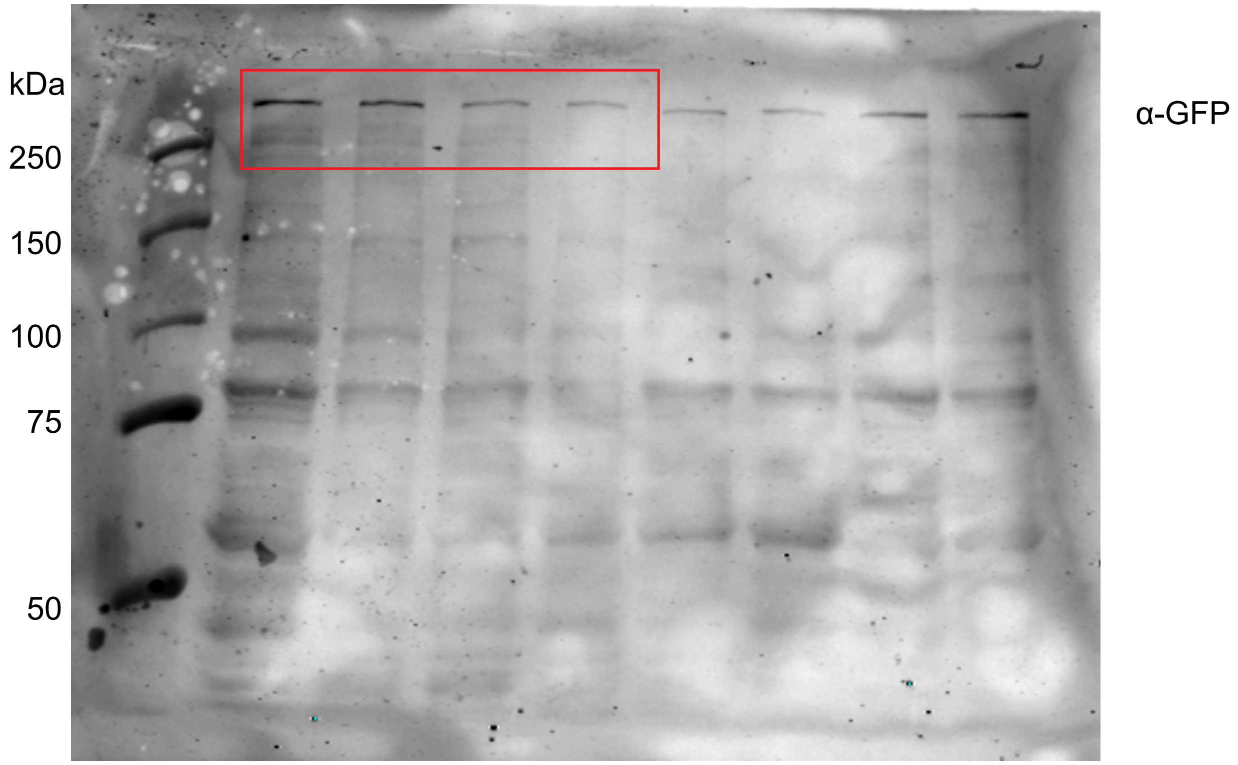

Supplementary Fig. 1d

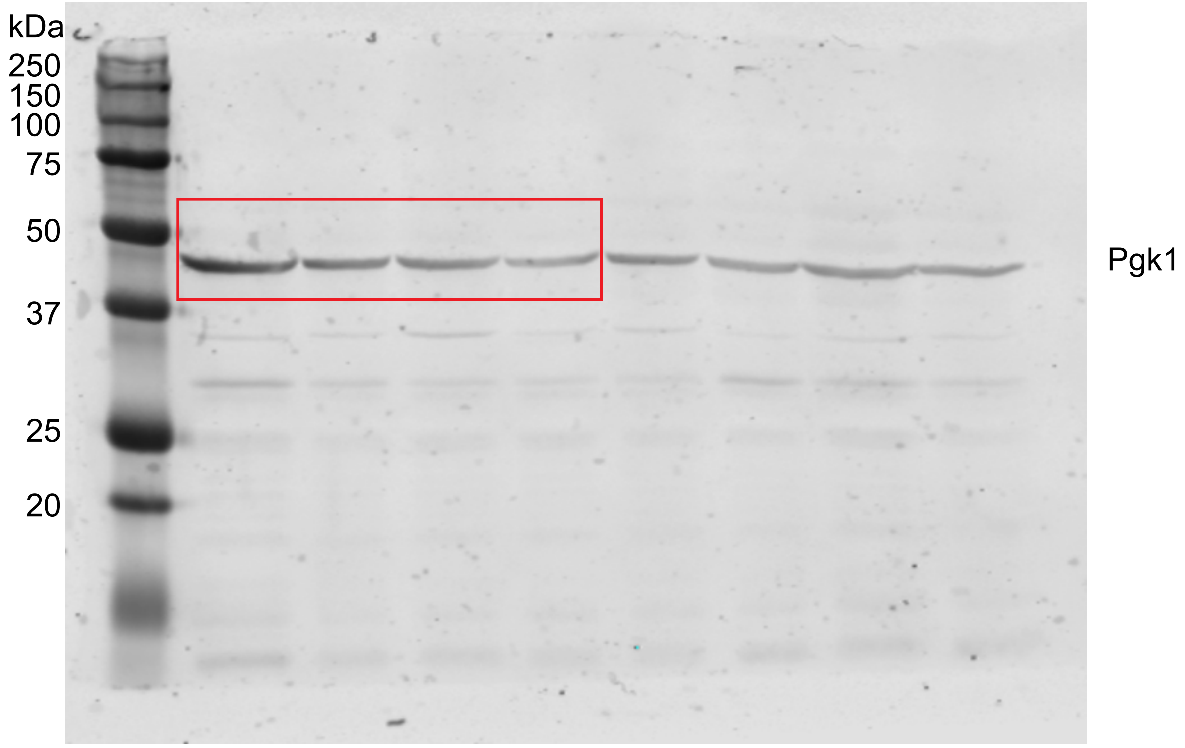

Supplementary Fig. 1g

Supplementary Fig. 7a

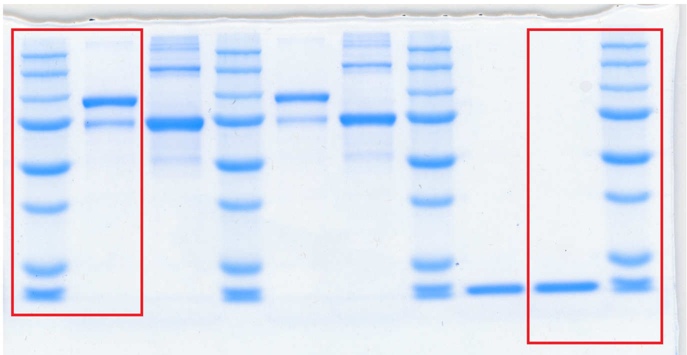

Supplementary Fig. 5b (Supernatant fraction)

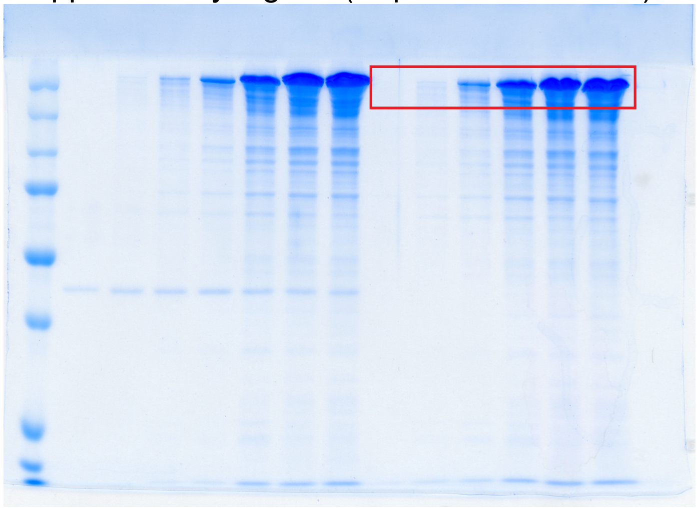

Supplementary Fig. 5b (Pellet fraction)

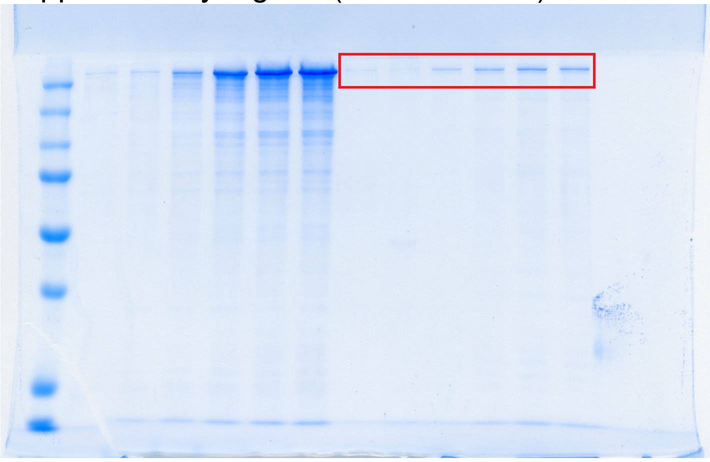

Supplementary Fig. 5h

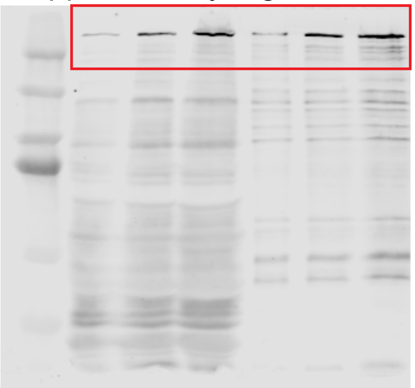

Supplementary Fig. 5h

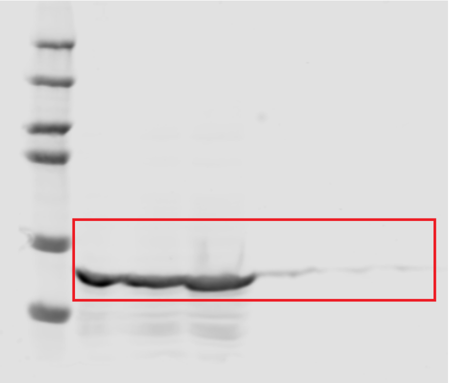

Supplementary Fig. 6c

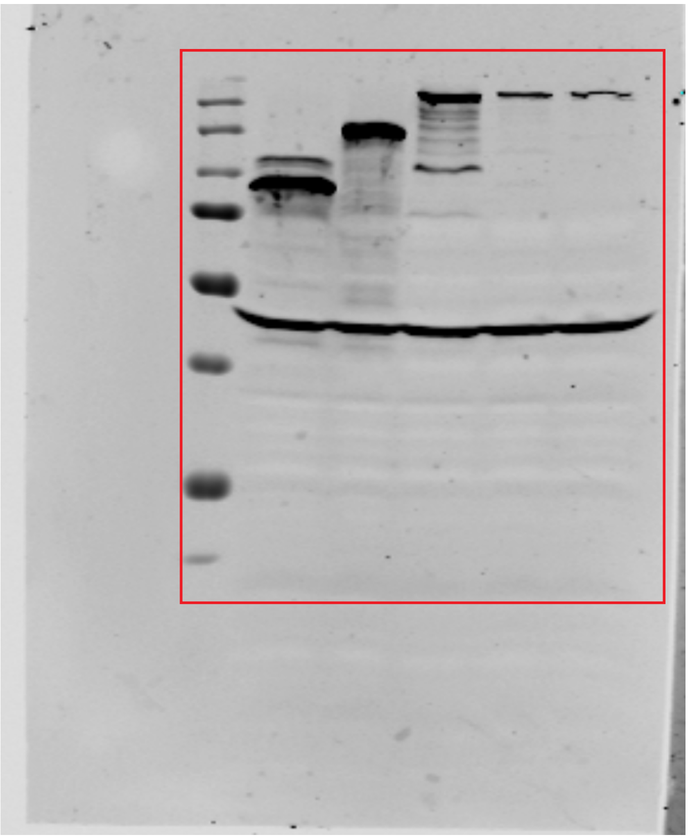

Supplementary Fig. 6d

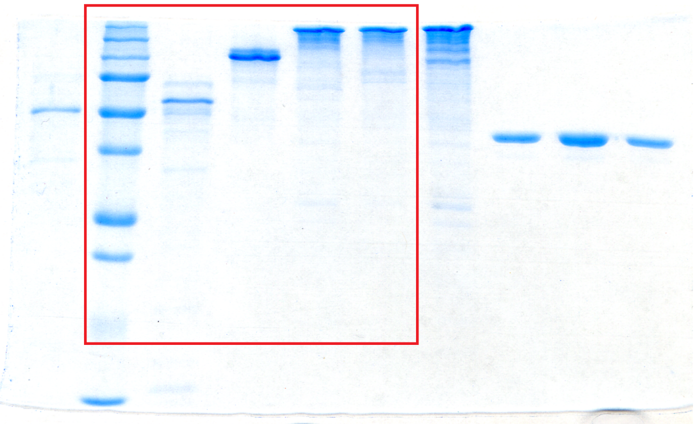

Supplementary Fig. 7d

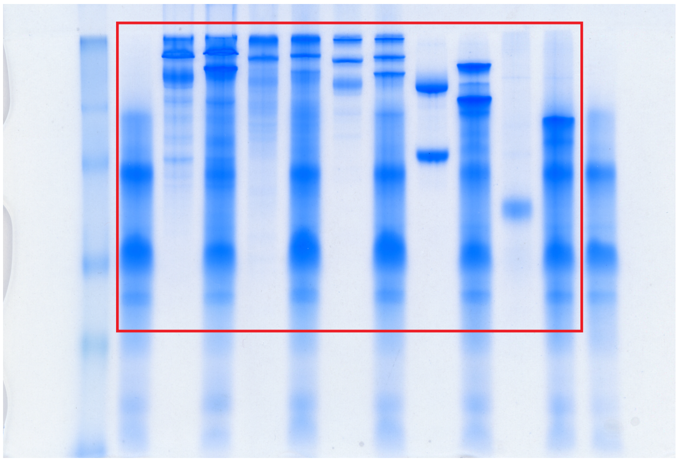

Supplementary Table 1. Yeast strains used in the study.

Strain name	Genotype	Source
YMY2011	MATa/MATα *his3-Δ200/his3-Δ200 ura3-52/ura3-52 leu2-3,112/leu2-3,112 lys2-801*/LYS2 *ade2-1*/ADE2	[1]
YMY2012	MATα *his3-Δ200 leu2-3, 112, ura3-52*	[1]
YMY2013	MATα *his3-Δ200 leu2-3, 112, ura3-52*, Aip5-GFP::KanMX	This study
YMY2014	MATα *his3-Δ200 leu2-3, 112, ura3-52, spa2*Δ::Leu2, Aip5-GFP::KanMX	This study
YMY2015	MATα *his3-Δ200 leu2-3, 112, ura3-52, bni1*Δ*::*Leu2, Aip5-GFP::KanMX	This study
YMY2016	MATα *his3-Δ200 leu2-3, 112, ura3-52, bud6*Δ*::*Ura3, Aip5-GFP::KanMX	This study
YMY2017	MATα *his3-Δ200 leu2-3, 112, ura3-52, spa2*Δ*::*Leu2, bni1Δ::His3, Aip5-GFP::KanMX	This study
YMY2018	MATa *his3-Δ200 leu2-3, 112, ura3-52*, Spa2-GFP::His3	This study
YMY2019	MATα *his3-Δ200 leu2-3, 112, ura3-52, Spa2*Δ*C*-GFP::KanMX4	This study
YMY2020	MATa *his3-Δ200 leu2-3, 112, ura3-52, Spa2*Δ*C*-GFP::KanMX4, Aip5-3mCherry::URA3	This study
YMY2021	MATα *his3-Δ200 leu2-3, 112, ura3-52*, Spa2-GFP::His3, Aip5-3mCherry::URA3	This study
YMY2022	MATα *his3-Δ200 leu2-3, 112, ura3-52*, Aip5-GFP::KanMX, Bni1-3mCherry::His3	This study
YMY2023	MATα *his3-Δ200 leu2-3, 112, ura3-52*, Aip5-GFP::KanMX, Bud6-3mCherry::His3	This study
YMY2024	MATa *his3-Δ200 leu2-3, 112, ura3-52*, Bni1-3GFP::His3 Bud6-3mCherry::His3 *cdk1-as1*::NAT	This study
YMY2025	MATα *his3-Δ200 leu2-3, 112, ura3-52*, Abp1-RFP::His3 Abp140-3GFP::His3	[1]
YMY2026	MATa *his3-Δ200 leu2-3, 112, ura3-52, aip5*Δ::Ura3, Abp1-RFP::His3 Abp140-3GFP::His3	This study
YMY2027	MATα *his3-Δ200 leu2-3, 112, ura3-52, bni1*Δ*::*Ura3, Abp1-RFP::His3 Abp140-3GFP::His3	[1]
YMY2028	MATα *his3-Δ200 leu2-3, 112, ura3-52, aip5*Δ*::*Ura3, *bni1*Δ*::*Ura3, Abp1-RFP::His3 Abp140-3GFP::His3	This study
YMY2029	MATα *his3-Δ200 leu2-3, 112, ura3-52, bnr1*Δ*::*KanMX, Abp140-3GFP::His3, Abp1-RFP::His3	This study
YMY2030	MATα *his3-Δ200 leu2-3, 112, ura3-52, aip5*Δ*::*Ura3 *bnr1*Δ*::*KanMX, Abp140-3GFP::His3, Abp1-RFP::His3	This study
YMY2031	MATα *his3-Δ200 leu2-3, 112, ura3-52, aip5-N*-GFP::Ura3	This study
YMY2032	MATα *his3-Δ200 leu2-3, 112, ura3-52, aip5*Δ*::*Ura3, *leu2-3:: aip5-C*-GFP::Leu2	This study
YMY2033	MATα *his3-Δ200 leu2-3, 112, ura3-52, aip5*Δ*::*Ura3, *leu2-3:: aip5-C*-GFP::Leu2, *spa2*Δ*::*Leu2	This study
YMY2034	MATα *his3-Δ200 leu2-3, 112, ura3-52, aip5-N*-GFP::Ura3, *spa2*Δ*::*Leu2	This study
YMY2035	MATa *his3-Δ200 leu2-3, 112, ura3-52, spa2*Δ*::*Leu2, Abp140-3GFP::His3	This study

YMY2036	MATα *his3-Δ200 leu2-3, 112, ura3-52, aip5-N*-GFP::Ura3, *bni1*Δ*::*Leu2, Abp140-3GFP::His3, Abp1-RFP::His3	This study
YMY2037	MATa *his3-Δ200 leu2-3, 112, ura3-52, aip5*Δ*::*Ura3, *bni1*Δ*::*Ura3, *leu2-3:: aip5-C*::Leu2, Abp140-3GFP::His3, Abp1-RFP::His3	This study
YMY2038	MATα *his3-Δ200 leu2-3, 112, ura3-52, Aip5*-13Myc::KanMX	This study
YMY2039	MATα *his3-Δ200 leu2-3, 112, ura3-52, aip5-N*-13Myc::KanMX	This study
YMY2040	MATα *his3-Δ200 leu2-3, 112, ura3-52, aip5-N1014*-13Myc::KanMX	This study
YMY2041	MATα *his3-Δ200 leu2-3, 112, ura3-52, aip5-N457*-13Myc::KanMX	This study
YMY2042	MATα *his3-Δ200 leu2-3, 112, ura3-52, aip5-N271*-13Myc::KanMX	This study
YMY2043	Mata *ade2, leu2, his,3 trp1, ura3, lys2::Pgal1:GAL4::Lys2, pep4::His3, bar1::HisG*	[1]
YMY2044	MATα *his3-Δ200 leu2-3, 112, ura3-52, aip5*Δ*::*Ura3	This study

Supplementary Table 2 Oligonucleotide primers used in the study.

Aip5-GFP/13Myc-F	AAGAAGCTAACGAAGACTACAAATTGCGGGAACTCATTTAC GATACTATTCGGATCCCCGGGTTAATTAA
Aip5-GFP/13Myc-R	ACATGTGCTGTCAAGAGATTATGTTAGTTACGTAGCGCGCTT AAGCTTTCGAATTCGAGCTCGTTTAAAC
Aip5-N-GFP/13Myc-F	CTACTGAGCAATCCAAGAAAAACAATGACAAGCCACAAGAC GTAATAACGCGGATCCCCGGGTTAATTAA
Spa2-C-GFP-F	AAGTGAAGATGACGATGAAGAGGAAGAAGACAGTGATTTTG ATCGGATCCCCGGGTTAATTAA
Spa2-C-GFP-R	CTTTGTCTTCCTTTTCTTTCTCCTCTAGATACTACTAACTGAA TTCGAGCTCGTTTAAAC
P634-Aip5-F	TTAATTAATGGTGCAACCG
P634-Aip5N-R	CGTTATTACGTCTTGTGG
P634-Aip5N1014-R	AGGAAGAGAAGTTTCTTTCTC
P634-Aip5N457-R	ACCGTGTTGTAGATTTTTTTC
P634-Aip5N271-R	GTTAGGCACATCTATGTC
Aip5-N1014-13Myc-F	ATTGGAAGTAGAAGAAACAGAGAAAGAAACTTCTCTTCCTC GGATCCCCGGGTTAATTAA
Aip5-N1014-13Myc-R	CATTCTTTTCCTCCGTGATGTTTTCTTCCACTACAAGATCGAA TTCGAGCTCGTTTAAAC
Aip5-N457-13Myc-F	AAAGCGCATTAGTGACAATGAAAAAAATCTACAACACGGTC GGATCCCCGGGTTAATTAA
Aip5-N457-13Myc-R	CTTCTTCTTCTTTTTCAACTTCTACAGATATATCGTTAGTGAA TTCGAGCTCGTTTAAAC
Aip5-N271-13Myc-F	TGAATCTAGTGACAAAACCTTCGACATAGATGTGCCTAACCG GATCCCCGGGTTAATTAA
Aip5-N271-13Myc-R	TGTTCTCGGACTTTGAGGAAGTTTCGTCAACATTATCTTTGA ATTCGAGCTCGTTTAAAC
Bni1FH2C-F	TTTGAAAAGTATCCTCGTCCACAC
Bni1FH2C-R	CATGGATTGGAAGTACAGGTTCTC
Spa2-LC-Vec-F	TGACAGTAAAGGTGGATACGG
Spa2-LC-Vec-R	CATGGATTGGAAGTACAGGTTCTC

Spa2-LC-Insert-F	CTGTACTTCCAATCCATGACTGGTGCATATACGAAAAC
Spa2-LC-Insert-R	CGTATCCACCTTTACTGTCACTTCAACTTCGAATTCAAATAA TT

References

1. Miao, Y. *et al.* Fimbrin phosphorylation by metaphase Cdk1 regulates actin cable dynamics in budding yeast. *Nature communications* **7**, 11265 (2016).
